# Structural characterization and subcellular localization of *Drosophila* organic solute carrier partner 1

**DOI:** 10.1186/1471-2091-15-11

**Published:** 2014-06-18

**Authors:** Nguyen Tho Huu, Hideki Yoshida, Takanari Umegawachi, Seiji Miyata, Masamitsu Yamaguchi

**Affiliations:** 1 Department of Applied Biology, Matsugasaki, Sakyo-ku, Kyoto 606-8585, Japan; 2 Insect Biomedical Research Center, Kyoto Institute of Technology, Matsugasaki, Sakyo-ku, Kyoto 606-8585, Japan

**Keywords:** Organic solute carrier partner 1, *Drosophila*, Subcellular organelle

## Abstract

**Background:**

Organic solute carrier partner 1 (OSCP1) is known to facilitate the transport of various organic solutes into cells and reported to play a role in cell growth and cell differentiation. Moreover, OSCP1 is known as a tumor suppressor gene that is frequently down-expressed in nasopharyngeal carcinomas and acute myeloid leukemia. However, the underlying mechanisms of action remain unclear and the subcellular localization of OSCP1 has yet to be determined in detail.

**Results:**

*Drosophila* contains a single orthologue of OSCP1 (dOSCP1) that shares 58% homology with its human counterpart. To study the expression pattern and subcellular localization of dOSCP1, we prepared a specific antibody. Subcellular localization analyses of dOSCP1 with these revealed localization in the plasma membrane, endoplasmic reticulum, Golgi apparatus and mitochondria, but no detection in cytosol. dOSCP1 signals were also detected in the nucleus, although at weaker intensity than in plasma membranes and subcellular organelles. In addition, native polyacrylamide gel electrophoresis analysis with and without β-mercaptoethanol treatment revealed that recombinant dOSCP1 forms dimers and trimers in solution. The dimer form of dOSCP1 could also be detected by Western immunoblot analyses in third instar larval extracts.

**Conclusions:**

The data revealed that dOSCP1 localizes not only in the plasma membrane but also in the nucleus, ER, Golgi apparatus and mitochondria. It is therefore conceivable that this protein may interact with various partners or form multimeric complexes with other proteins to play multiple roles in cells, providing clues to understanding the functions of dOSCP1 during *Drosophila* development.

## Background

Transporter proteins play important roles in absorption and elimination of numerous endogenous molecules, as well as exogenous substances and their metabolites, from cells. Moreover, identification and characterization of transporter proteins are important for drug discovery and development [1]. The mechanisms involved in cellular transport of organic anions and cations have been studied and reviewed elsewhere [2-4]. Transporter proteins belong to two major superfamilies: solute carrier (SLC) and ATP-binding cassette (ABC) [5]. The SLC transporters include two families consisting of the organic anion transporting polypeptides (OATPs) and the *SLC22A* group, which contains the organic cation transporters (OCTs) and the organic anion transporters (OATs) [6]. In general, transporters are designed to recognize a single substance or a group of very similar substances, although some carrier proteins such as OATPs show broad substrate specificities [6,7].

Recently, organic solute carrier protein 1 (OSCP1) was identified in mammals as a polyspecific solute carrier protein [8-11] and likely novel member of the SLC transporters. When expressed in *Xenopus laevis* oocytes, OSCP1 mediated high affinity transport of p-aminohippurate (PAH), tetraethylammonium, and a wide range of structurally diverse organic compounds including prostagladin E2, prostaglandin F2α, estron sulfate, glutarate, L-leucine, L-ascorbic acid and tetracycline [8-11]. These results suggest that OSCP1 mediates transport of various organic solutes into cells.

On the other hand the *OSCP1* gene, also named as *Oxidored-nitro domain-containing protein 1* (*NOR1*), has been identified as a tumor suppressor gene that is frequently down-expressed in nasopharyngeal carcinomas (NPCs) [12-16]. Moreover, the *OSCP1* (*NOR1*) promoter region has been found to be frequently methylated in acute myeloid leukemia (AML) patients [17]. These data indicate that OSCP1 may play a role in genesis of NPC and AML, although the underlying mechanisms are not fully understood.

Most SLC22A super family proteins are predicted to consist of 12 transmembrane domains, with intracellular amino and carboxy-termini [18,19], although organic solute transporter alpha (OSTα) and beta (OSTβ) contain 7 and 1 transmembrane domains, respectively [20]. However, the structure of OSCP1 has not yet been biochemically and physically characterized. In the present study, we therefore examined the structure of *Drosophila melanogaster* OSCP1 (dOSCP1). Native polyacrylamide gel electrophoresis analysis with and without β-mercaptoethanol treatment revealed that the recombinant dOSCP1 forms dimers and trimers in solution. The dimer form of dOSCP1 was further confirmed by Western immunoblot analyses with third instar larval extracts.

Subcellular localization of OSCP1 is controversial. Although it has been reported to localize in plasma membranes of human trophoblast cells and mouse Sertoli cells [9,11], cytoplasmic localization has also been reported in mouse cerebral neuronal cells [8,21] and human HeLa cells [14]. Therefore, in this study, we examined subcellular localization of dOSCP1 and revealed its presence in the plasma membrane, endoplasmic reticulum, Golgi apparatus, mitochondria and nucleus of cells. The data indicate that dOSCP1 plays not only in the transport of organic solutes through the cell membrane, but also into the organelles and nucleus, and consequently it may be involved in regulation of apoptosis, differentiation and/or proliferation.

## Results

### OSCP1 is an evolutionary conserved protein across species

**Figure 1:**
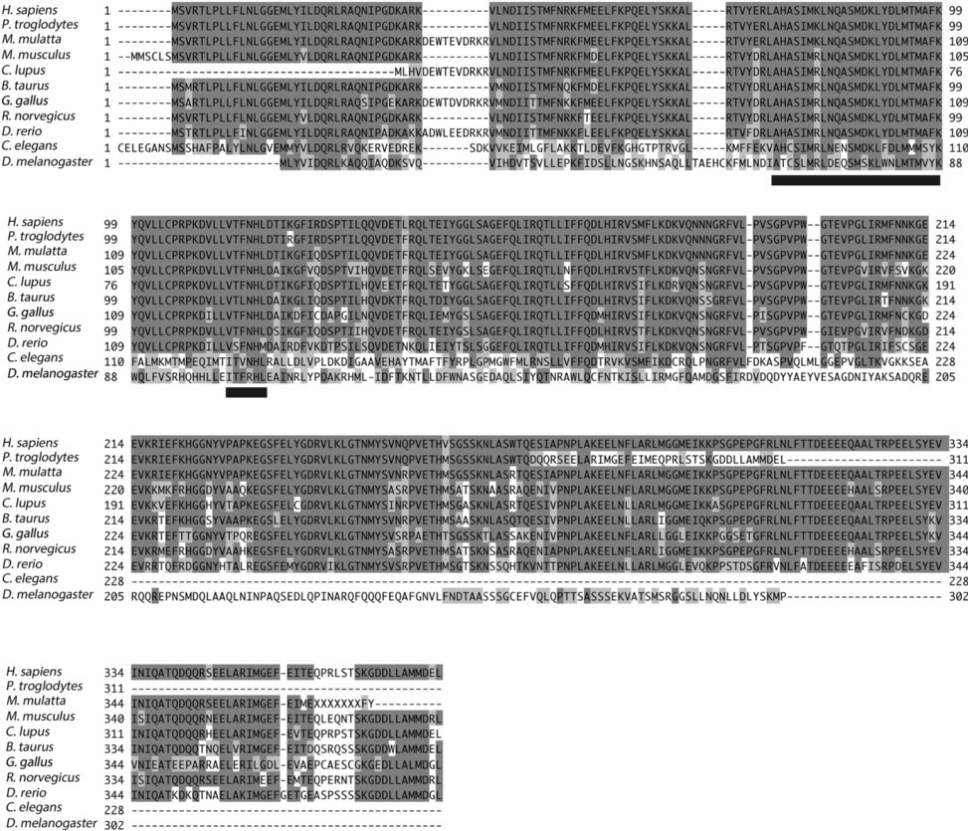
**OSCP1 is a protein conserved in evolution across multiple species*.*** Sequence alignment of OSCP1 proteins from different species. Identical amino acids are displayed with black shading and similar amino acids are in gray. The highly conserved regions are underlined.

We used the NCBI database (http://www.ncbi.nlm.nih.gov/) to access information on OSCP1. The data showed that the *OSCP1* gene is conserved among multiple species such as *H.sapiens, P.troglodytes, M.mulatta, M.musculus, C.lupus, B.taurus, G.gallus, R.norvegicus, D.rerio, C.elegans and D.melanogaster,* but not in *S.cerevisiae*. To gain insight into the relationships of the OSCP1 among individual species, sequence alignments were analyzed by ClustalW2 software. Identical amino acid residues are shaded in black and similar amino acid residues are in gray in Figure 1, and the highly conserved regions 1 and 2 are underlined. The similarity and identity of amino acid sequences of human OSCP1 (hOSCP1) and *Drosophila* (dOSCP1, CG13178) were found to be 58% and 30%, respectively. The most highly conserved region 1 of hOSCP1 and dOSCP1 (aa63 to aa87) showed 92% similarity and 48% identity. The highly conserved region 2 of these two proteins (aa103 to aa108) showed 100% similarity and 57% identity. These two conserved regions may thus play important roles in OSCP1 function, although further analyses are necessary to clarify this point.

### Expression and purification of recombinant dOSCP1 protein

**Figure 2:**
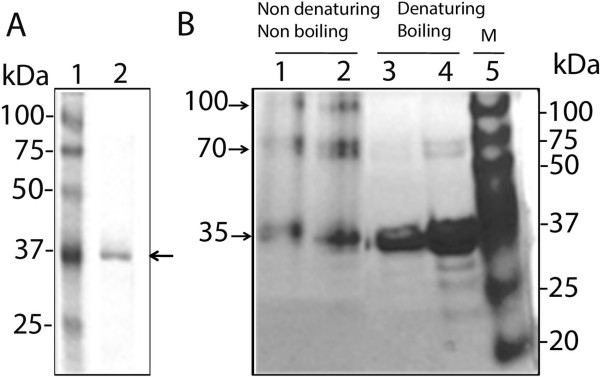
**dOSCP1 forms dimers and trimers in solution. (A)** Purified recombinant His-dOSCP1 protein was separated on SDS-PAGE containing 10% acrylamide. The gel was then stained with Coomassie Brilliant Blue G-250. Lane 1: Precision protein standards (Bio-Rad) with molecular weights given in kilodaltons. Lane 2: purified His-dOSCP1 protein. The arrow indicates the 35 kDa band. **(B)**. Native PAGE analysis of the recombinant dOSCP1. Lanes 1 and 2: 2 μg or 4 μg of protein were treated with sample buffer containing no SDS, no β-mercaptoethanol and without boiling. The samples were separated on Native-PAGE containing 10% acrylamide. The gel was then stained with Coomassie Brilliant Blue G-250. The bands detected were at 35 kDa, 70 kDa and 100 kDa band. The 35 kDa band corresponds to His-dOSCP1 recombinant protein, the 70 kDa band likely represents the dimeric form and the 100 kDa band may represent a trimeric form. Lanes 3 and 4: 2 μg or 4 μg of protein were treated with SDS sample buffer containing 4% SDS and 10% β-mercaptoethanol in boiling water. Lane 3: the 70 kDa band is not detectable. Land 4: A faint 70 kDa band is evident. Lane 5: Precision protein standards (Bio-Rad) with molecular weights given in kilodaltons (kDa).

The expression plasmid pCold-dOSCP1 and empty vector pColdI were transformed into the *E. coli* BL21 strain. Expression of the recombinant His-dOSCP1 fusion protein was induced by adding 0.5 mM IPTG at 18°C for 15 hours. Most recombinant protein was found in the soluble fraction (data not shown) and Ni-NTA method was used to purify the His-dOSCP1 fusion protein for analysis by sodium dodecyl sulfate-polyacrylamide gel electrophoresis (SDS-PAGE). This latter detected a single 35 kDa band on Coomassie Brilliant Blue G-250 (CBB) staining (Figure 2A). The dOSCP1 protein contains 302 amino acids and the calculated molecular weight is 34,607, that is nearly identical in size to the detected band. The purity of the recombinant dOSCP1 in the final fraction was estimated to be more than 95%. Western immunoblot analysis with monoclonal anti-His antibody showed the 35 kDa band to truly represent the His-dOSCP1 (data not shown).

### Structural analysis of dOSCP1 by native PAGE

To evaluate the possible multimer formation of dOSCP1, we performed native-PAGE analysis of recombinant dOSCP1 protein, treated with or without 4% SDS and 10% β-mercaptoethanol with or without heating. The apparent dimer form of dOSCP1 was detected at the position corresponding to 70 kDa when compared with the standard maker (Figure 2B, lanes 1 and 2). In addition a more slowly migrating band corresponding to 100 kDa was also detected that could represent a trimer form of dOSCP1 under the native PAGE conditions. In any event, by this method, we first established that dOSCP1 can form multimers in solution.

**Figure 3:**
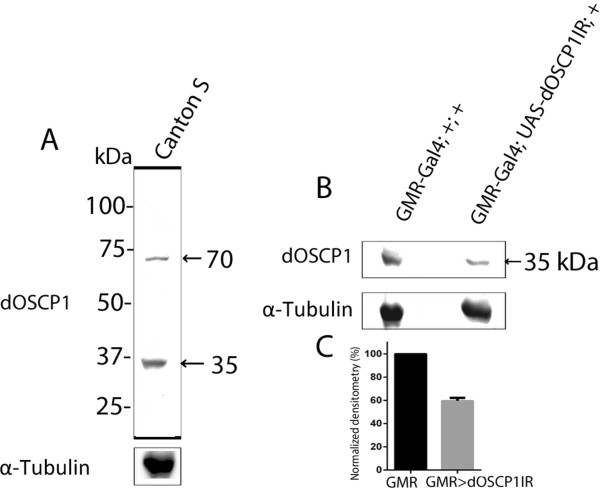
**Western immunoblot analysis. (A)** Proteins extracted from Canton S third instar larval whole bodies. The blots were probed with a Guinea pig polyclonal anti-OSCP1 antibody. In the immunoblotting, both 35 kDa and 70 kDa bands were detected. The 35 kDa band corresponds to dOSCP1 protein. The faint 70 kDa band likely represents the dimeric form. **(B)** Protein extracts of eye imaginal discs from GMR-Gal4; +; + (control flies) and GMR-Gal4; UAS-*dOSCP1IR*; + (knockdown flies). Knockdown of dOSCP1 led to decrease in the 35 kDa band when compared with control flies. **(C)** Quantification of the 35 kDa band using Image Saver 6 software. The relative expression level was significantly decreased in knockdown flies (*P < 0.001; Student t test; n = 3; 30 μg of proteins per replicate). α-Tubulin was used as a loading control.

Western immunoblot analysis with Canton S third instar larval extracts using anti-dOSCP1 antibody revealed a 35 kDa dOSCP1 band (Figure 3A). The intensity of this was decreased by 40% in extracts of eye discs in which dOSCP1 double stranded RNA (dsRNA) was specifically expressed with GMR-GAL4 and UAS-*dOSCP1IR* (Figure 3B-C). The results thus indicated that the 35 kDa band truly represents the dOSCP1 protein. The faint 70 kDa band for the putative dimer form of dOSCP1 was also detected even under denaturing conditions with 4% SDS and 10% β-mercaptoethanol (Figure 3A), suggesting that dOSCP1 forms dimers *in vivo*. Dimer or multimer formation in the presence of SDS has been reported for *Drosophila* β-sarcoglycan [22] and mammalian sarcoglycans [23]. It has also been reported that some cell surface antigens such as HLA-DR, -DC1 and –B7 form dimeric or multimeric forms even with SDS-PAGE [24,25].

### Subcellular localization of dOSCP1 protein in various Drosophila larval tissues

**Figure 4:**
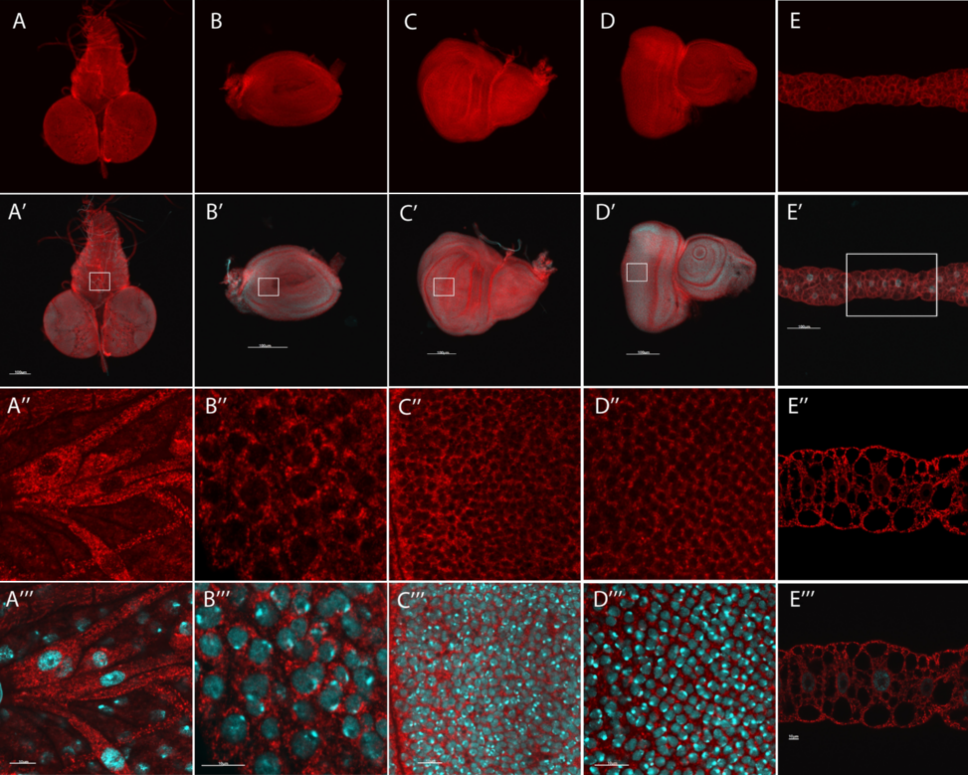
**Ubiquitous expression dOSCP1 in various tissues.** Brain lobe, fat body and several imaginal discs of third instar larvae immunostained with anti-dOSCP1 antibody. **(A)** brain lobe, **(B)** leg disc, **(C)** wing disc, **(D)** eye disc, **(E)** fat body. Microscopic inspections were carried out under the same conditions for the immunostaining with the antibody preincubated with the purified His-dOSCP1 fusion protein (Additional file 1: Figure S1). **(A”-E”)** higher magnification images of the indicated regions of the upper panels. **(A’-E’)** and **(A”’-E”’)** Merged images of several imaginal discs with DAPI for nuclear staining (Blue) and anti-dOSCP1 antibody (Red). The bars indicate 100 μm **(A’-E’)** or 10 μm **(A”’-E”’)**.

**Figure 5:**
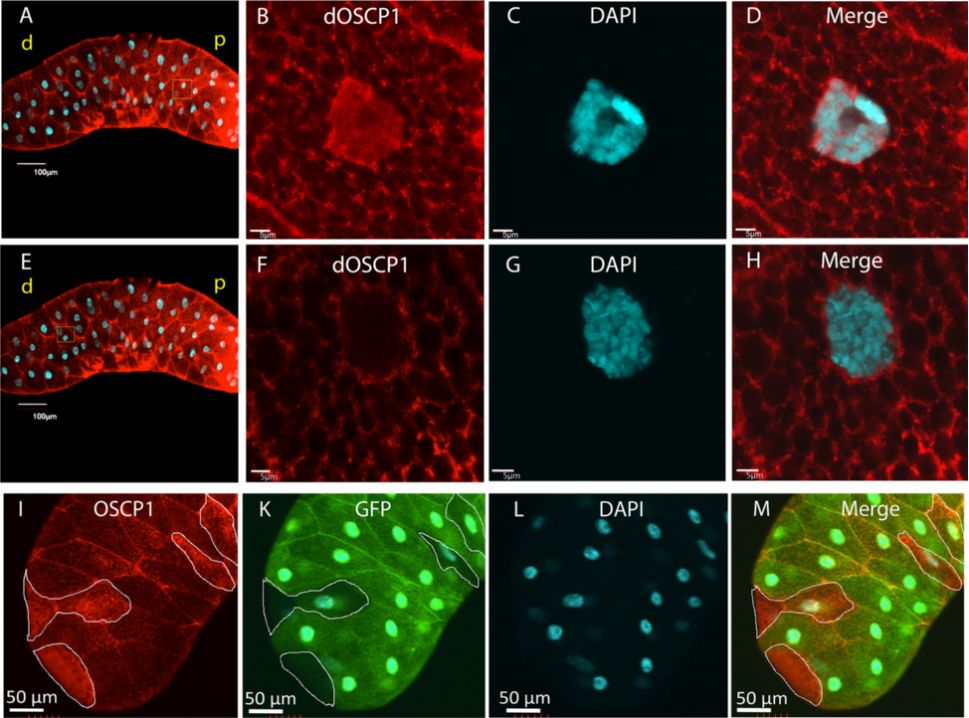
**Localization of dOSCP1 in salivary gland cells.** Salivary glands dissected from third instar larvae were stained with anti-dOSCP1 antibody and DAPI. **(A-D)** Image of proximal region. **(A)** Merged image of dOSCP1 signals and DAPI. **(B-D)** High magnification images of the proximal region. **(B)** dOSCP1 signals, **(C)** DAPI, **(D)** Merged image. A significant level of dOSCP1 signals was detected in the nuclei. **(E-H)** Image of distal region. **(E)** Merged image of dOSCP1 signal and DAPI. **(F-H)** High magnification images of the distal region. **(F)** dOSCP1 signals, **(G)** DAPI, **(H)** Merged image. No signals of dOSCP1 are evident in nuclei. **(I-M)** Image of flip-out expreriment. **(I)** OSCP1. **(K)** GFP. **(L)** DAPI. **(M)** Merged image. GFP-negative areas are enclosed. In the GFP-positive dOSCP1 RNAi clone area, levels of dOSCP1 signals are reduced. The bars indicate 100 μm **(A; E)** or 50 μm **(B-D and I-M)** or 5 μm **(F-H)**. p, proximal; d, distal.

The goal of our study was molecular, structural, and functional characterization of dOSCP1 protein localized in cells. As a first step towards this goal, the anti-dOSCP1 antibody was used to examine the expression pattern of the dOSCP1 protein in various tissues in the third instar larvae of *Drosophila*. Immunostaining results showed dOSCP1 to be expressed ubiquitously in various tissues including brain lobes, leg discs, wing discs, eye discs, fat bodies (Figure 4) and salivary glands (Figure 5). No signals were detected with the primary dOSCP1 antibody preincubated with the purified His-dOSCP1 fusion protein (Additional file 1: Figure S1), indicating specificity for the OSCP1 protein. Immunohistochemical results showed that dOSCP1 signals were mainly detectable in the cytoplasm as dotted or mesh-like structures (Figure 4A’-E”). Salivary glands showed strong dOSCP1 signals in the cell membrane and in a cytoplasmic mesh-like structure (Figure 5A-H). In the proximal regions of the glands, significant signals of dOSCP1 were also detected in the nucleus (Figure 5A-D). In contrast, no nuclear signals were detectable in the distal region (Figure 5E-H).To further evaluate the signals with anti-dOSCP1 antibody, a flip-out experiment was employed to make a somatic clone expressing dOSCP1 dsRNA in the salivary glands. In RNAi clones marked with GFP, the level of dOSCP1 signals was specifically reduced (Figure 5I-M), thus confirming that the anti-dOSCP1 signals both in the cytoplasm and nucleus truly represent dOSCP1 protein.

### Localization of dOSCP1 protein on plasma membranes

**Figure 6:**
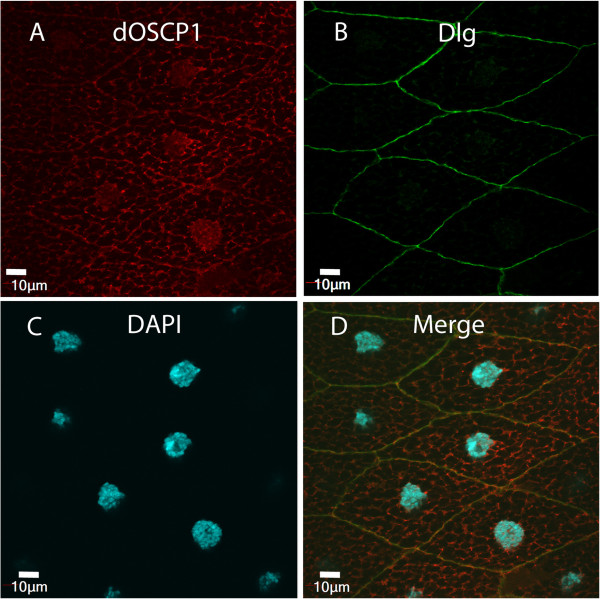
**Plasma membrane localization of dOSCP1.** Colocalization of dOSCP1 with Dlg. Salivary glands of third instar larvae were stained with anti-dOSCP1 **(A)**, anti-Dlg (plasma membrane marker) **(B)** antibodies and DAPI **(C)**. **(D)** Merged image. The bars indicate 10 μm.

To confirm the localization of dOSCP1 in the cell membrane, we performed double-immunostaining assay with anti-dOSCP1 antibody and anti-Discs large (Dlg) antibody. Dlg has been reported as a component of septate junction structures in *Drosophila* epithelia cells [26]. Anti-Dlg antibody labels cell membranes and was therefore used here as a marker. Immunostaining showed signals with the anti-dOSCP1 antibody to overlap those with the anti-Dlg antibody (Figure 6).

### Localization of dOSCP1 protein in subcellular organelles

In immunostaining with salivary gland, we observed dOSCP1 signals in a mesh-like structure in the cytoplasm (Figure 5). As a first step to clarify the nature of this dOSCP1-positive structure, regarding the precise localization of the OSCP1 protein in the cytoplasm, double-immunostaining of the salivary gland with anti-dOSCP1 and specific antibodies for each organelle was performed.

**Figure 7:**
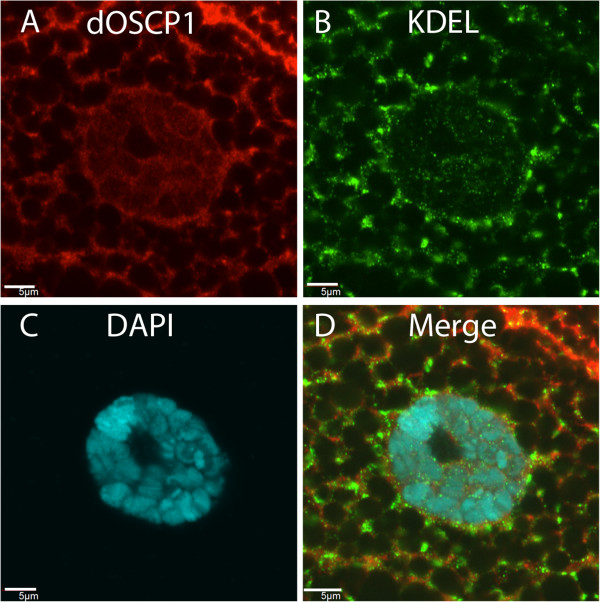
**ER localization of dOSCP1.** Salivary glands of third instar larvae coimmunostained with anti-dOSCP1 and anti-KDEL antibodies **(A-D)**. DAPI was used to stain nuclei. The bars indicate 5 μm.

To investigate the localization of dOSCP1 in the endoplasmic reticulum (ER), we used anti-dOSCP1 antibody and anti-KDEL antibody for double immunostaining of the third instar larval salivary gland. The anti-KDEL antibody has been used as the ER marker [27] which labels the sequence Lys-Asp-Glu-Leu (KDEL), or a closely related sequence, that is present at the carboxyl terminus of ER soluble and some membrane proteins [28-31]. Double-immunostaining showed co-localization of dOSCP1 and KDEL signals (Figure 7A-D). It is reported that in the salivary gland, KDEL signals are detectable not only in ER but also in cell and nuclear membranes [27]. This staining pattern with the anti-KDEL antibody appears to be intrinsic to this highly secreting tissue, since immunostaining of the cultured S2 cells with the same antibody showed signals in ER without any detectable signals in cell and nuclear membranes (Additional file 2: Figure S2). These results indicate that dOSCP1 localizes in ER.

**Figure 8:**
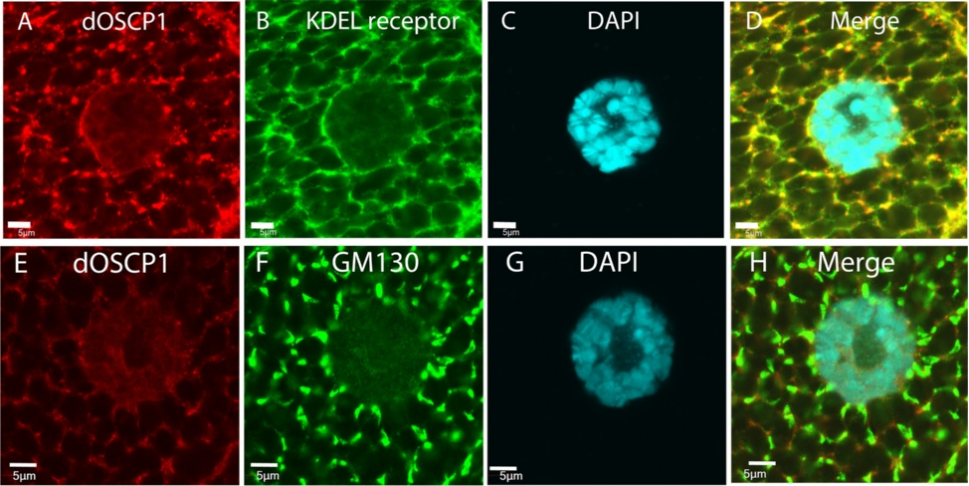
**Golgi apparatus localization of dOSCP1.** Salivary gland third instar larvae stained with anti-dOSCP1 **(A)**, anti-KDEL receptor antibodies (upper panels) **(A-D)**, or with anti-dOSCP1 and anti-GM130 antibodies (Golgi apparatus marker) (lower panels) **(E-H)**. DAPI was used to stain nuclei. The bars indicate 5 μm.

To examine the localization of dOSCP1 in the Golgi apparatus, we employed double labeling with anti-dOSCP1 and anti-KDEL receptor antibodies. KDEL receptor is an integral membrane protein which mediates the retrieval of solute resident proteins from Golgi apparatus to the ER [28,32-34]. Some signals with anti-KDEL receptor in cell and nuclear membranes in addition to Golgi apparatus observed in this analysis are also reported with the salivary gland [27]. The results showed co-localization of dOSCP1 and KDEL receptor signals (Figure 8A-D).

To further evaluate the Golgi apparatus localization of dOSCP1, double immunostaining with anti-dOSCP1 and anti-GM130 antibodies was performed. The anti-GM130 antibody detects a peripheral membrane protein GM130 that binds to the Golgi complex [35]. The data showed overlapping signals (Figure 8E-H), further confirming the Golgi apparatus localization of dOSCP1.

**Figure 9:**
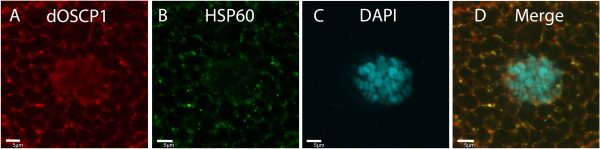
**Mitochondria localization of dOSCP1.** Salivary glands of third instar larvae stained with anti-dOSCP1 **(A)**, anti-Heat shock protein 60 (HSP60) (mitochondria marker) **(B)** antibodies and DAPI **(C). (D)** Merged image. DAPI was used to stain nuclei. The bars indicate 5 μm.

In addition, localization of dOSCP1 in mitochondria was examined. To test this, the mitochondria protein HSP60 [36] was labeled with anti-HSP60 antibody. Double immunostaining with anti-dOSCP1 and anti-HSP60 antibodies showed the co-localization in cytoplasmic dots (Figure 9A-D). Thus a mitochondrial localization of dOSCP1 was also demonstrated.

## Discussion

OSCP1 has been reported to mediate the transport of various organic solutes such as organic anions and cations across the plasma membrane in a pH-dependent and sodium-independent manner [8-10]*.* However, the underlying mechanisms of OSCP1 function have been unclear. Up to now, there has been no report on the structure of OSCP1 protein. Previous studies demonstrated that oligomerization of many transporter proteins plays an important role in movement of materials across cells [37-39]. Thus, oligomerization allows proteins to form large structures without increasing genome size and provides stability, while the reduced surface area of monomers in a complex can offer protection against denaturation [18,40,41]. Moreover, many proteins are known to self-assemble into oligomers to perform their biological functions [42]. In this study we found that dOSCP1 could form dimers and trimers in solution, which may be required for its transporter functions. Regarding membrane transporter proteins, dimerization and higher order oligomerization have actually been proposed as important factors that modulate their activity [43]. Therefore, further investigation of the dimerization of dOSCP1 would appear necessary to ascertain the underlying mechanisms of function.

In addition, several membrane transporters such as OATs are predicted to have a 12 membrane spanning domain [44], while the exceptions of OSTα and OSTβ predicted to have seven and one, respectively [20]. We used bioinformatics tools to gain insight into whether or not *Drosophila* OSCP1 carries a potential transmembrane domain(s). However, Kyte-Doolittle hydropathy analysis method indicated that *Drosophila* OSCP1 carries no potential transmembrane domain (http://gcat.davidson.edu/DGPB/kd/kyte-doolittle.htm). The same prediction was obtained with the SMART program (http://smart.embl-heidelberg.de/smart/set_mode.cgi?NORMAL=1) and the TMpred program (http://www.ch.embnet.org/software/TMPRED_form.html). Furthermore, a previous study predicted that mouse OSCP1 does not carry any potential transmembrane domain [8]. These observations indicate that dOSCP1 may be not imbedded in the membrane but associated with some membrane bound proteins and consequently indirectly mediate and facilitate the transport of organic solutes into the cells. It is well established that many proteins associated with the cell membrane are either directly or indirectly involved in membrane transport [45-47]. Multimeric complex formation was also reported for L-type amino acid transporter 1 with the 4EF2 heavy chain [48], and between OSTα and OSTβ [20]. Taken together these observations suggest that dOSCP1 may associate with other transporters to assist their functions. Future investigations should focus on whether OSCP1 acts as a regulator or a constituent of the functional transporter system in cells.

Various membrane transporters have been detected in different tissues including the blood–brain barrier, lung, heart, intestine and kidney [44]. The broad tissue distribution of human, mouse and rat OSCP1 has been well characterized, brain, lung, trachea, spinal chord and nasopharynx being included as shared sites [9,11,13,14]. Therefore, the present detection of dOSCP1 in various *Drosophila* larval tissues including brain lobes, eye discs, wing discs, leg discs, fat body and salivary glands suggests that the broad distribution pattern of OSCP1 is conserved among multiple species.

In addition to the dotted or mesh-like structures of dOSCP1 signals in the cytoplasm of the salivary glands, we detected some nuclear dOSCP1 signals in the proximal regions of the glands. In contrast, no nuclear signal was detectable in the distal region. It is known that most of cells in salivary glands of late stage of third instar larvae have completed polytenization in the distal region, but not in the proximal region [49]. Therefore dOSCP1 may play a role in nuclei of endoreplicating cells. Although dOSCP1 does not contain a canonical nuclear localization signal (NLS), the PSORT II tool based on the amino acid sequence predicts nuclear localization of dOSCP1 with 76.7% reliability (http://psort.hgc.jp/form2.html). However, since we cannot exclude the possibility that the antibody may be more accessible to the proximal region, further analyses are necessary to confirm these observations.

## Conclusions

In the present study, we could show that dOSCP1 localizes not only in the plasma membrane but also in the nucleus, ER, Golgi apparatus and mitochondria. It is therefore conceivable that this protein may interact with various partners or form multimeric complexes with other proteins to play multiple roles in cells. Whatever the case, the present data should help in furthering understanding of the roles of the *dOSCP1* gene during *Drosophila* development.

## Methods

### Sequence comparison of the OSCP1 gene

The OSCP1 homologue sequence from the GeneBank database was analyzed in different species: *(Homo sapiens)* OSCP1 (NP_659484.4), *(Pan troglodytes)* OSCP1 (JAA38622.1), *(Macaca mulatta)* OSCP1 (XP_002802362), *(Mus musculus)* OSCP1 (NP_766289.2), *(Canis lupus)* OSCP1 (XP_53256.3), *(Bos Taurus)* OSCP1 (NP_001039369.1), *(Gallus gallus)* OSCP1 (XP_001233001.1), *(Rattus norvegicus)* OSCP1 (NP_001025094.1), *(Danio rerio)* OSCP1 (XP_003201654.1), *(Caenorhabditis elegans)* R10F2.5 (NP_497640.1), *(Drosophila melanogaster)* CG13178 (AFF58577.1). Sequence alignment was performed using ClustalW2 software.

### Oligonucleotides

For construction of pCold-dOSCP1, the following oligonucleotides were synthesized.

dOSCP1FXhoI: 5’-CTCGGTACCCTCGAGATGCTCTACGTGATCGATCAG-3’

dOSCP1REcoRI: 5’-GACAAGCTTGAATTCTCAAGGCATTTTGCTGTACAG-3’

### Plasmid construction

To construct the plasmid pCold-dOSCP1, the full length cDNA of dOSCP1 was amplified by PCR with primers carrying XhoI and EcoRI sites and the amplified fragment was inserted into the plasmid pColdI (Takara, Japan) using an Infussion HD Kit (ClonTech, Japan)

### Preparation of the recombinant dOSCP1-His fusion protein and production of anti-dOSCP1 antibody

The amplified full length dOSCP1 cDNA carrying XhoI and EcoRI sites was cloned into the pColdI vector (Takara, Japan) to carry a C terminal His tag fusion sequence. After the nucleotide sequence was confirmed by sequencing, the plasmid was transformed into *E. coli* BL21. Expression of His-dOSCP1 fusion protein was carried out with a cold shock expression system (Takara, Japan). Cell lysates were prepared by sonication in PBS containing 1 mM phenylmethylsulfonyl fluoride (PMSF) and 1% SDS, then the supernatant and the pellets were separated by centrifugation at 12,000 *g* for 20 min at 4°C. The supernatant was diluted with binding buffer (500 mM NaCl, 50 mM NaH_2_PO4, pH 8.0, 5 mM Imidazol, 1 mM PMSF) and purified with Ni-NTA agarose (QIAGEN, USA). After purification, the His-dOSCP1 fusion proteins were used to immunize a Guinea pig (SLC, Inc. Japan). Production of polyclonal antibodies was checked by Western blotting.

### Fly stocks

Fly stocks were maintained at 25°C on standard food containing 0.7% agar, 5% glucose and 7% dry yeast. The transgenic fly line carrying glass minimal response element GMR-GAL4 on X chromosome was as described previously [50]. Fly lines carrying UAS-*dOSCP1IR*_*466–609*_ were obtained from the Vienna *Drosophila* RNAi Center.

### Flip out experiments

RNAi clones in *Drosophila* larval salivary gland were generated with a flip-out system [51]. Female flies with hs-flp; Act5C > FRT y FRT > Gal4, UAS-GFP were crossed with male flies carrying UAS-*dOSCP1IR*_*466–609*_*.* Clones were evaluated by the presence of green fluorescent protein (GFP) expressed under control of the *Act5C* promoter. Flip-out was induced by heat shock (60 min at 37°C) at 24–48 hours after egg laying.

### Immunohistochemistry

For immunohistochemistry, third instar larvae were dissected and fixed in 4% paraformaldehyde/PBS for 15 min at 25°C. After washing with PBS/0.3% Triton X-100 (PBS-T), the samples were blocked with PBS containing 0.15% Triton X-100 and 10% normal goat serum for 20 min at 25°C and incubated with primary antibodies for 16 h at 4°C. The following antibodies were used: anti-dOSCP1 (1:100), mouse anti-Dlg (1:400) (Developmental Studies of Hybridoma Bank, DSHB), mouse anti-KDEL (1:800) (Enzo Life Sciences), mouse anti-KDEL receptor (1:300) (Abcam), rabbit anti-GM130 (1:200) (Abcam), mouse anti-HSP60 (1:200) (Enzo Life Sciences). For negative control, 10 μg of Guinea pig IgG dOSCP1 antibody was incubated with 15 μg of the purified His-dOSCP1 fusion protein for 16 h at 4°C and used as primary antibody (1:100). After washing with PBST, samples were incubated with secondary antibodies labeled with either Alexa 594 or Alexa 488 (1:400; Invitrogen, Tokyo, Japan) for 3 hours at 25°C. Following further washing with PBS/0.3% Triton X-100 and with PBS, the samples were stained with DAPI (0.5 μg/ml)/PBS/0.1% Triton X-100. After washing with PBST and PBS, the samples were mounted and observed under a confocal laser scanning microscope (OLYMPUS Fluoview FV10i).

### Native-PAGE analysis

Two μg and 4 μg of His-dOSCP1 fusion protein were prepared in a non denaturing sample buffer (62.5 mM Tris–HCl pH 6.8, 25% Glycerol, 1% bromophenol blue) on ice [52] and denaturing sample buffer (10% β-mercaptoethanol, 4% sodium dodecyl sulfate, 20% glycerol, 0.25% bromophenol blue, 0.125 M Tris–HCl pH 6.8) in boiling water for 5 min [52]. Proteins were separated on polyacrylamide gels containing 10% acrylamide without SDS at a constant voltage of 100 V for 6 hours at 4°C [52]. The gels were then stained with Coomassie Brilliant Blue G-250 (CBB).

### SDS-PAGE and Western immunoblot analysis

Eye imaginal discs of third instar larvae of wild type and transgenic flies carrying GMR-Gal4; UAS-dOSCP1IR; + were dissected in PBS and homogenized in an extraction buffer containing 50 mM Tris–HCl (pH7.5), 5 mM MgCl2, 150 mM NaCl, 10% glycerol, 0.1% Triton X-100, 0.1% NP-40, 10 μg/ml each of aprotinin, leupeptin and pepstatin A and 1 μg/ml each of antipain, chymostatin and phosphoramidon. Homogenates were centrifuged and total protein quantified by the Bradford method [53]. 30 μg of proteins were boiled at 100°C for 5 min, electrophoretically separated on SDS-polyacrylamide gels containing 10% acrylamide and then transferred to polyvinylidene difluoride membranes (Bio-Rad, USA). The membranes were blocked in TBS-0.05% Tween 20 containing 5% skim milk for 1 hour at 25°C, followed by incubation with mouse monoclonal anti-His antibody (DSHB) at 1:1500 or Guinea pig polyclonal anti-dOSCP1 antibody at 1:1500 dilution or mouse monoclonal anti-α-tubulin antibody (DSHB) at 1:5000 for 16 hours at 4°C. After washing 5 times with TBS, the membranes were incubated with HRP-conjugated rabbit anti-Guinea pig IgG (H + L) (Invitrogen, USA) at 1:10,000 dilution or stabilized peroxidase-conjugated goat anti-mouse (H + L) (Thermo Scientific, USA) at 1:5,000 dilution for 1 hour at 25°C. Detection was performed with ECL Western blotting detection reagents (GE healthcare, USA). Images were captured by AE-9300H Ez–Capture MG system (ATTO corporation, Japan) and analyzed with Image Saver6 software (ATTO corporation, Japan).

### Availability of supporting data

The data sets supporting the results of this article are available in the LabArchves. The unique persistent identifier and hyperlink to data sets in https://mynotebook.labarchives.com/share/Masamitsu%2520Yamaguchi/MzIuNXwzOTQ2OS8yNS9UcmVlTm9kZS8xNjQ2ODMxOTAxfDgyLjU= (Additional file 1: Figure S1).

https://mynotebook.labarchives.com/share/Masamitsu%2520Yamaguchi/MzYuNHwzOTQ2OS8yOC9UcmVlTm9kZS8yODcxNjUyNTgyfDkyLjQ= (Additional file 2: Figure S2).

## Abbreviations

OSCP1: Organic solute carrier partner 1; SLC: Solute carrier; ABC: ATP-binding cassette; OATPs: Organic anion transporting polypeptides; OCTs: Cation transporters; OATs: Organic anion transporters; PAH: P-aminohippurate; NOR1: Oxidored-nitro domain-containing protein 1; AML: Acute myeloid leukemia; NPCs: Nasopharyngeal carcinomas, signal; CBB: Coomassie brilliant blue G-250; NLS: Nuclear localization signal; UAS: Upstream activation sequence; Dlg: Discs large; ER: Endoplasmic reticulum; HSP60: Heat shock protein 60; SDS-PAGE: Sodium dodecyl sulfate-polyacrylamide gel electrophoresis; BPB: Bromophenol blue; dsRNA: Double strand RNA.

## Competing interests

The authors declare that they have no competing interests.

## Authors’ contributions

NTH conducted the experiments, analyzed the data. SM designed and performed the experiments to produce anti-dOSCP1 antibodies. TU performed the experiments for revision. NTH, HY and MY conceived and designed the study, edited and wrote the manuscripts. All authors read and approved the final manuscript.

## Supplementary Material

Additional file 1: Figure S1 Identification of the absorption of anti-dOSCP1 antibody and His-dOSCP1 fusion proteins. Brain lobe, fat body and several imaginal discs of third instar larvae immunostained with anti-dOSCP1 antibody preincubated with the purified His-dOSCP1 fusion proteins. Microscopic inspections were carried out under the same conditions for the immunostaining with the antibody without preincubation (Figure 4). (A) brain lobe, (B) leg disc, (C) wing disc, (D) eye disc, (E) fat body, (F) salivary gland. (A”-F”) higher magnification images of the indicated regions of the upper panels. (A’-F’) and (A”’-F”’) Merged images of several imaginal discs with DAPI (Blue) and anti-dOSCP1 antibody (Red). The bars indicate 100 μm (A’-F’) or 10 μm (A”’-F”’). Click here for file

Additional file 2: Figure S2 Localization of KDEL, GM130 and KDEL receptor in cultured *Drosophila* S2 cells. Cells stained with anti-KDEL (A), anti-GM130 (B), anti-KDEL receptor (C) antibodies, (D) absence of the primary antibodies (Green) and DAPI (Blue). (A’-D’) merged images. The bars indicate 10 μm. Click here for file
