# Supplementary figures and images for: Structural characterization and subcellular localization of Drosophila organic solute carrier partner 1
Source: BMC Biochem. 2014 Jun 18;15:11. doi: 10.1186/1471-2091-15-11 (PMC4074837; doi:10.1186/1471-2091-15-11)

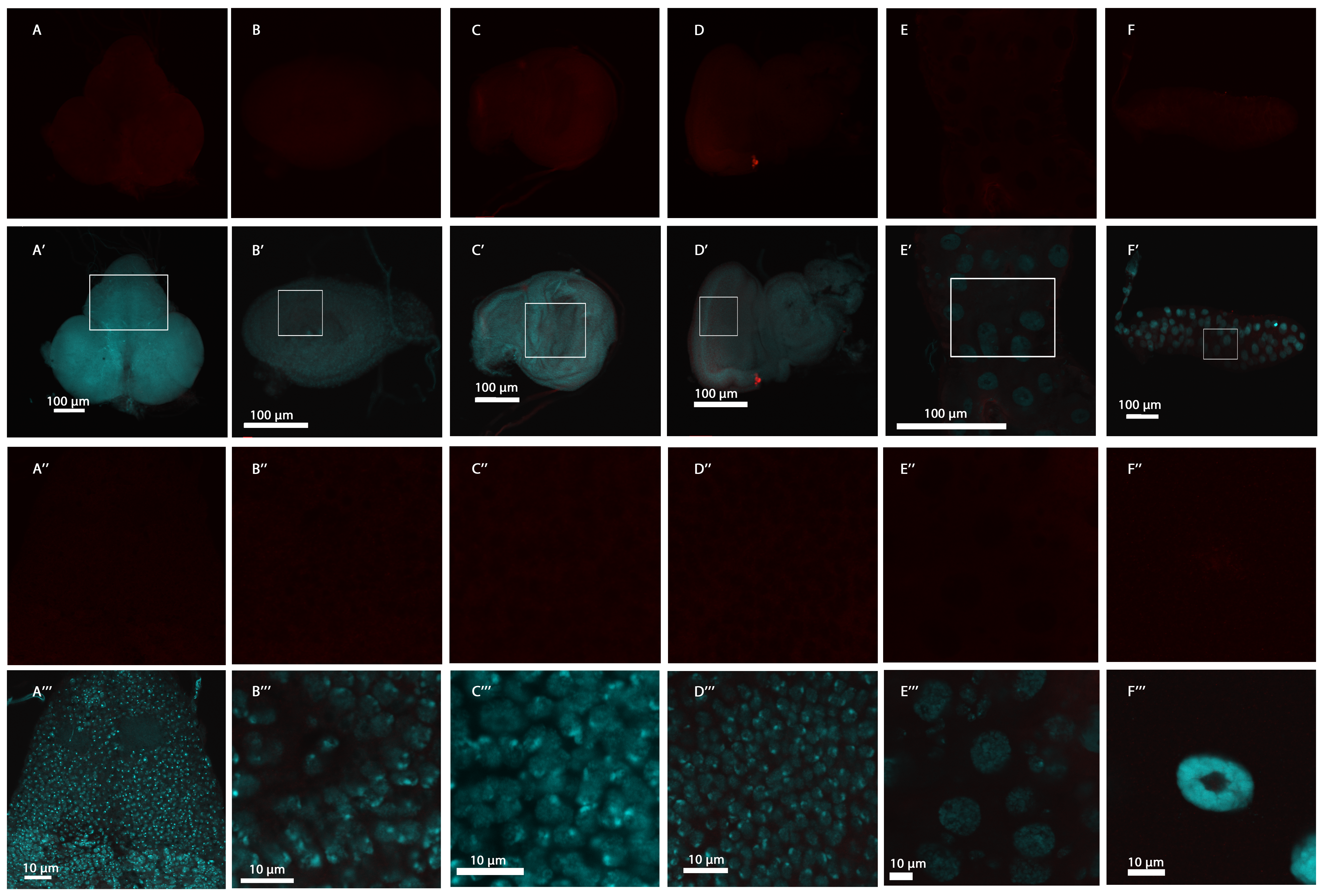

Supplement: Additional file 1: Figure S1 — Identification of the absorption of anti-dOSCP1 antibody and His-dOSCP1 fusion proteins. Brain lobe, fat body and several imaginal discs of third instar larvae immunostained with anti-dOSCP1 antibody preincubated with the purified His-dOSCP1 fusion proteins. Microscopic inspections were carried out under the same conditions for the immunostaining with the antibody without preincubation (Figure 4). (A) brain lobe, (B) leg disc, (C) wing disc, (D) eye disc, (E) fat body, (F) salivary gland. (A”-F”) higher magnification images of the indicated regions of the upper panels. (A’-F’) and (A”’-F”’) Merged images of several imaginal discs with DAPI (Blue) and anti-dOSCP1 antibody (Red). The bars indicate 100 μm (A’-F’) or 10 μm (A”’-F”’). [file 1471-2091-15-11-S1.tiff]

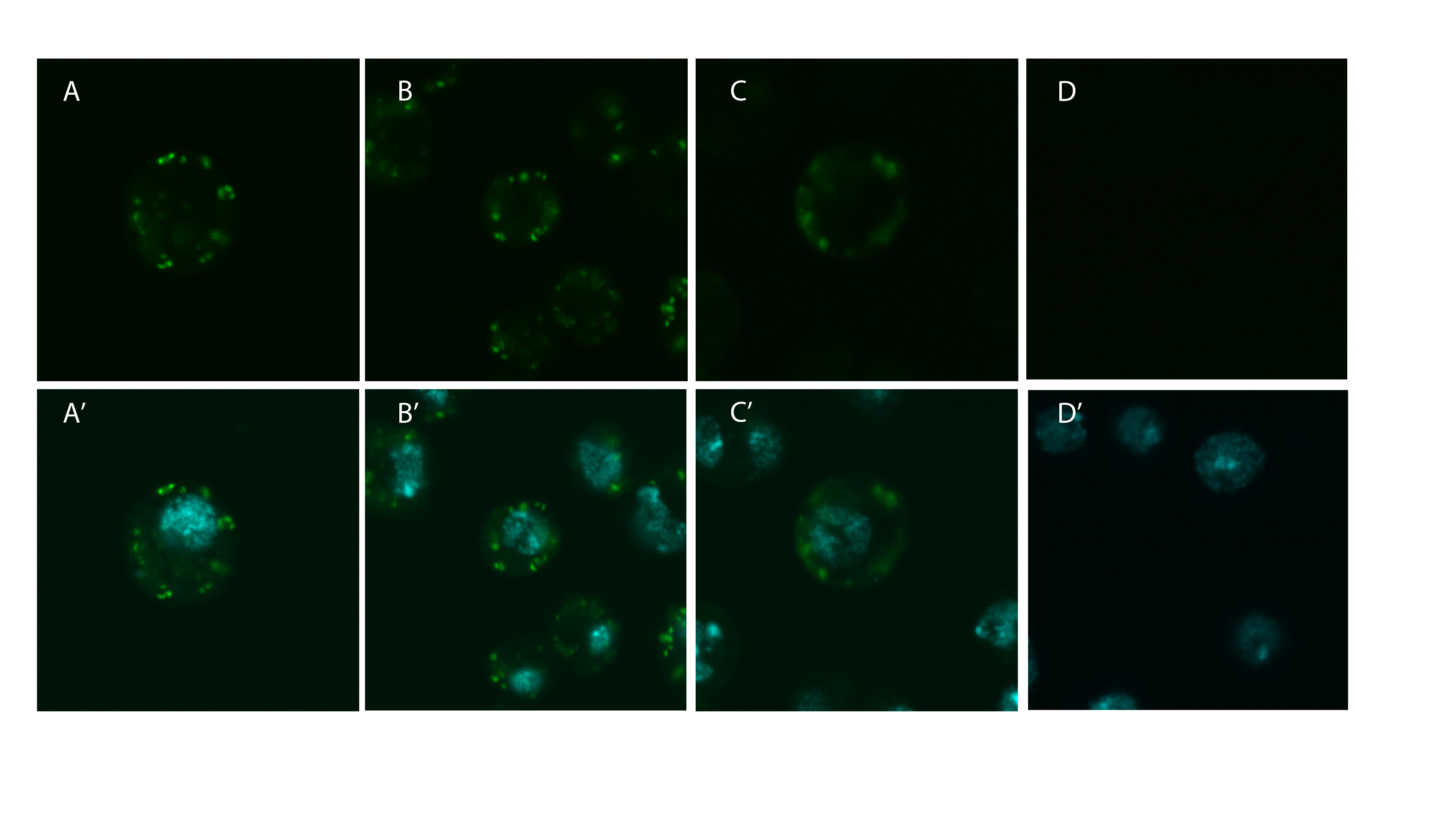

Supplement: Additional file 2: Figure S2 — Localization of KDEL, GM130 and KDEL receptor in cultured Drosophila S2 cells. Cells stained with anti-KDEL (A), anti-GM130 (B), anti-KDEL receptor (C) antibodies, (D) absence of the primary antibodies (Green) and DAPI (Blue). (A’-D’) merged images. The bars indicate 10 μm. [file 1471-2091-15-11-S2.tiff]
